# The therapeutic potential of exercise and caffeine on attention-deficit/hyperactivity disorder in athletes

**DOI:** 10.3389/fnins.2022.978336

**Published:** 2022-08-12

**Authors:** Abigail S. Sogard, Timothy D. Mickleborough

**Affiliations:** Department of Kinesiology, School of Public Health-Bloomington, Indiana University, Bloomington, IN, United States

**Keywords:** caffeine, exercise, performance, attention-deficit/hyperactivity disorder, adult ADHD

## Abstract

Attention-deficit/hyperactivity disorder (ADHD) is characterized by evident and persistent inattention, hyperactivity, impulsivity, and social difficulties and is the most common childhood neuropsychiatric disorder, and which may persist into adulthood. Seventy to 80% of children and adults with ADHD are treated with stimulant medication, with positive response rates occurring for both populations. Medicated ADHD individuals generally show sustained and improved attention, inhibition control, cognitive flexibility, on-task behavior, and cognitive performance. The ethics of ADHD medication use in athletics has been a debated topic in sport performance for a long time. Stimulants are banned from competition in accordance with World Anti-Doping Association and National Collegiate Athletic Association regulations, due to their ability to not only enhance cognitive performance but also exercise performance. Limited research has been conducted looking at the differences in exercise performance variables in unmedicated ADHD verses medicated ADHD. Not all ADHD athletes choose stimulant medication in their treatment plan due to personal, financial, or other reasons. Non-stimulant treatment options include non-stimulant medication and behavioral therapy. However, the use of caffeinated compounds and exercise has both independently been shown to be effective in the management of ADHD symptoms in human studies and animal models. This mini review will discuss the effect of exercise and caffeine on neurobehavioral, cognitive, and neurophysiological factors, and exercise performance in ADHD athletes, and whether exercise and caffeine should be considered in the treatment plan for an individual with ADHD.

## Introduction

Attention-deficit/hyperactivity disorder (ADHD) is characterized by evident and persistent inattention and hyperactivity-impulsivity and is the most common childhood neuropsychiatric disorder and may persist into adulthood in 30–65% of patients, whereby symptoms of inattentiveness appear to outweigh those of hyperactivity (Konrad and Eickhoff, 2010; Minzenberg, 2012; Volkow and Swanson, 2013; White et al., 2014; Chabreck, 2015; Choi et al., 2015; Poysophon and Rao, 2018; Pretus et al., 2018; Stewman et al., 2018; Mehren et al., 2019; Cipollone et al., 2020; Stibbe et al., 2020; Berezanskaya et al., 2022; Vázquez et al., 2022; Ágoston et al., 2022). Within the general population, ADHD affects 2–7% of adults and 4–8% of the elite athlete population (Minzenberg, 2012; Volkow and Swanson, 2013; White et al., 2014; Choi et al., 2015; Poysophon and Rao, 2018; Pretus et al., 2018; Stewman et al., 2018; Cipollone et al., 2020; Stibbe et al., 2020; Berezanskaya et al., 2022; Ágoston et al., 2022). The Diagnostic and Statistical Manual of Mental Disorders: Fifth Edition (DSM-5) is the most up-to-date diagnostic tool for ADHD (Volkow and Swanson, 2013; White et al., 2014; Chabreck, 2015; Poysophon and Rao, 2018; Stewman et al., 2018). The DSM-5 focuses on symptoms present at the time of assessment, resulting in two symptom categories: hyperactivity/impulsivity and inattention (Volkow and Swanson, 2013; White et al., 2014; Chabreck, 2015; Poysophon and Rao, 2018; Stewman et al., 2018). Symptoms of inattention includes lacking attention overall, attention to detail, and difficulty organizing tasks to be completed (Volkow and Swanson, 2013; White et al., 2014; Chabreck, 2015; Poysophon and Rao, 2018; Stewman et al., 2018). Symptoms of hyperactivity/impulsivity include difficulties sitting still, especially for long periods, and excessive behavior like overtalking, interrupting, or being loud (Volkow and Swanson, 2013; White et al., 2014; Chabreck, 2015; Poysophon and Rao, 2018; Stewman et al., 2018). The age at which symptoms of ADHD began has been modified from 7-years old and younger to 12-years old and younger in the DSM-5 (Volkow and Swanson, 2013; White et al., 2014; Chabreck, 2015; Poysophon and Rao, 2018; Stewman et al., 2018). Adults over the age of 17 must present 5 out of 9 symptoms from either category for a positive ADHD diagnosis (Volkow and Swanson, 2013; White et al., 2014; Chabreck, 2015; Poysophon and Rao, 2018; Stewman et al., 2018). This mini review will discuss the effect of exercise and caffeine on neurobehavioral, cognitive, and neurophysiological factors, and exercise performance in ADHD individuals, and whether exercise and caffeine should be considered in the treatment plan for an individual with ADHD.

## The neurobiology of attention-deficit/hyperactivity disorder

The neurobiology of ADHD are multifaceted and involve functional changes in connectivity within the brain such as frontostriatal, frontoparietal, frontocerebellar, parieto-occipital, and cingulate cortex pathways, as well as dysregulation in the dorsolateral prefrontal cortex and the default motor network (Smith, 2003; Wang et al., 2009; Wolf et al., 2009; Konrad and Eickhoff, 2010; Minzenberg, 2012; Volkow and Swanson, 2013; Chabreck, 2015; Choi et al., 2015; Pretus et al., 2018; Mehren et al., 2019). Decreased activity has been shown repeatedly in executive function networks, attentional networks, and deactivation of the default motor network in cognitive tasks that are responsible for reduced inhibition control, organization, concentration, problem-solving, working memory, fluency, and reinforcers (Smith, 2003; Wang et al., 2009; Wolf et al., 2009; Konrad and Eickhoff, 2010; Minzenberg, 2012; Volkow and Swanson, 2013; Chabreck, 2015; Choi et al., 2015; Pretus et al., 2018; Mehren et al., 2019). Wolf et al. (2009) found that during working memory tasks, ADHD adults showed lower connectivity between the bilateral ventrolateral prefrontal cortex, the anterior cingulate cortex, the superior parietal lobe, and the cerebellum. Others have demonstrated that dysregulation of functioning networks resulted in decreased glucose metabolism and decreased cerebral blood flow (CBF) in frontal lobes during cognitive tasks contributing to decrements in executive function and increased ADHD symptoms (Shaywitz et al., 1983; Zametkin and Rapoport, 1987; Lou et al., 1989; Zametkin et al., 1990; Smith, 2003; Tan et al., 2020). Additionally, a significant negative relationship was found between cerebral blood flow and ADHD symptoms (Tan et al., 2020). Furthermore, dysregulation in the dorsal striatal and ventral dopamine (DA) systems and norepinephrine (NE) systems may play a major role in the pathophysiology of ADHD (Vallone et al., 2000; Smith, 2003; Madras et al., 2005; Forssberg et al., 2006; Spencer et al., 2007; Minzenberg, 2012; Volkow and Swanson, 2013; Kollins and Adcock, 2014; Chabreck, 2015; Choi et al., 2015; Sigurdardottir et al., 2016; Pretus et al., 2018; Stewman et al., 2018; Ranjbar-Slamloo and Fazlali, 2020). DA pathways start in the ventral tegmental area and project to many cortical areas involved in reward sensitivity, conscious movement, and emotion mediation (Vallone et al., 2000; Smith, 2003; Madras et al., 2005; Forssberg et al., 2006; Spencer et al., 2007; Minzenberg, 2012; Volkow and Swanson, 2013; Kollins and Adcock, 2014; Chabreck, 2015; Choi et al., 2015; Sigurdardottir et al., 2016; Pretus et al., 2018; Stewman et al., 2018; Ranjbar-Slamloo and Fazlali, 2020). DA metabolism is the synthesis and presynaptic release and uptake in neural networks. Synthesis begins with the amino acid tyrosine which is converted into L-dihydroxyphenylalanine (L-DOPA), then DA *via* DOPA-decarboxylase (Vallone et al., 2000; Smith, 2003; Madras et al., 2005; Forssberg et al., 2006; Spencer et al., 2007; Minzenberg, 2012; Volkow and Swanson, 2013; Kollins and Adcock, 2014; Chabreck, 2015; Choi et al., 2015; Sigurdardottir et al., 2016; Pretus et al., 2018; Stewman et al., 2018; Ranjbar-Slamloo and Fazlali, 2020). However, synthesis and storage rates are lower in ADHD and lower in the subcortical regions, which have reduced DA utilization (Vallone et al., 2000; Smith, 2003; Madras et al., 2005; Forssberg et al., 2006; Spencer et al., 2007; Minzenberg, 2012; Volkow and Swanson, 2013; Kollins and Adcock, 2014; Chabreck, 2015; Choi et al., 2015; Sigurdardottir et al., 2016; Pretus et al., 2018; Stewman et al., 2018; Ranjbar-Slamloo and Fazlali, 2020). NE systems extend to most of the central nervous system and are based in the locus coeruleus and lateral tegmental, involved in attention, response inhibition, memory, and autonomic function and motion (Vallone et al., 2000; Smith, 2003; Madras et al., 2005; Forssberg et al., 2006; Spencer et al., 2007; Minzenberg, 2012; Volkow and Swanson, 2013; Kollins and Adcock, 2014; Chabreck, 2015; Choi et al., 2015; Sigurdardottir et al., 2016; Pretus et al., 2018; Stewman et al., 2018; Ranjbar-Slamloo and Fazlali, 2020). NE is synthesized from DA *via* DA beta-hydroxylase and functions in the prefrontal cortex, acting on postsynaptic adrenergic receptors and presynaptic uptake (Vallone et al., 2000; Smith, 2003; Madras et al., 2005; Forssberg et al., 2006; Spencer et al., 2007; Minzenberg, 2012; Volkow and Swanson, 2013; Kollins and Adcock, 2014; Chabreck, 2015; Choi et al., 2015; Sigurdardottir et al., 2016; Pretus et al., 2018; Stewman et al., 2018; Ranjbar-Slamloo and Fazlali, 2020). However, in ADHD, a lack of NE ligands for the postsynaptic adrenergic receptors and transporters reduces overall functioning, resulting in core symptoms of ADHD.

## Pharmacology of attention-deficit/hyperactivity disorder

Approximately 25–78% of adults with ADHD show positive response rates with stimulant medication (Smith, 2003; Minzenberg, 2012; Volkow and Swanson, 2013; Chabreck, 2015; Stewman et al., 2018; Berezanskaya et al., 2022) and treatment has been shown to sustain and improve attention, inhibition control, cognitive flexibility, planning, verbal learning, on-task behavior, and cognitive performance (Smith, 2003; Minzenberg, 2012; Volkow and Swanson, 2013; Chabreck, 2015; Stewman et al., 2018; Berezanskaya et al., 2022). The most prescribed medications for ADHD are amphetamines (A; i.e., Adderall, Vyvanse) and methylphenidate (MPH; i.e., Concerta, Ritalin) (Smith, 2003; Minzenberg, 2012; Volkow and Swanson, 2013; Chabreck, 2015; Stewman et al., 2018; Berezanskaya et al., 2022). Immediate-release formulations allow for multiple doses within the day (four maximum) and last up to 3–4 h (Smith, 2003; Minzenberg, 2012; Volkow and Swanson, 2013; Chabreck, 2015; Stewman et al., 2018; Berezanskaya et al., 2022). Extended-release formulations allow for one dose that releases the medication throughout the day with effects lasting 6–10 h (Smith, 2003; Minzenberg, 2012; Volkow and Swanson, 2013; Chabreck, 2015; Stewman et al., 2018; Berezanskaya et al., 2022). Medication side effects include insomnia, dry mouth, decreased appetite, weight loss, headaches, depression, anxiety, and changes in resting heart rate and blood pressure (Smith, 2003; Minzenberg, 2012; Volkow and Swanson, 2013; Chabreck, 2015; Stewman et al., 2018; Berezanskaya et al., 2022). Stimulants act on attentional processes and increases response inhibition by crossing the blood-brain barrier to block the presynaptic reuptake of DA/NE (Smith, 2003; Minzenberg, 2012; Volkow and Swanson, 2013; Chabreck, 2015; Stewman et al., 2018; Berezanskaya et al., 2022).

**FIGURE 1:**
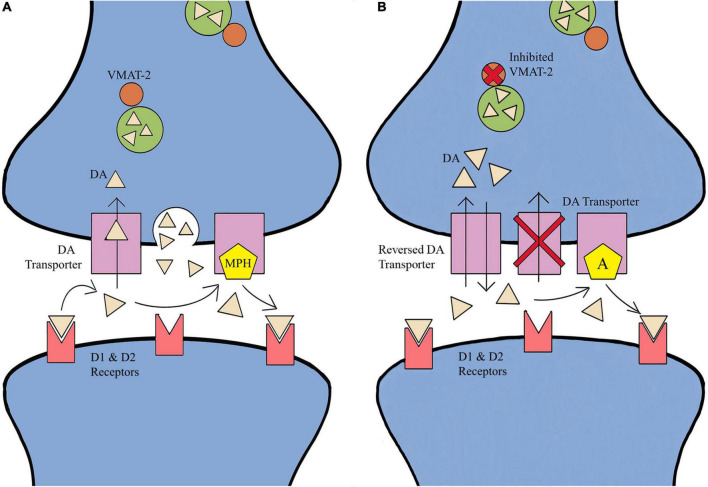
Effects of methylphenidate (MPH) and amphetamine (A) on neurotransmitter (NT) reuptake. **(A)** MPH inhibits NT transporters from reuptake into the presynaptic cell, increasing extracellular concentrations and increasing possible receptor binding. MPH treatment is shown to decrease NT transporter density in the striatum, thereby increasing the transportation of vesicular NT *via* vesicular monoamine transporter-2 (VMAT-2), leading to more NT within the synapse. **(B)** Amphetamine inhibits NT transporters in the reuptake of NT into the presynaptic cell and increases extracellular concentrations of NT in multiple brain regions. The increase in NT like dopamine concentrations within the synapse increases the incidence of receptor binding. Amphetamine is distinct from MPH in two ways: the inhibition of VMAT-2 results in the release if dopamine into the presynaptic terminal by presynaptic vesicles, and the internalization of a dopamine transporter resulting in the reversal of the transporter from in-flow to dual-flow of dopamine reuptake (Smith, 2003; Minzenberg, 2012; Volkow and Swanson, 2013; Chabreck, 2015; Stewman et al., 2018; Berezanskaya et al., 2022).

Methylphenidate inhibits DA/NE transporters (DAT and NET) from reuptake of DA/NE into the presynaptic cell, thereby increasing extracellular concentrations and increasing possible receptor binding (Figure 1). Additionally, activating adrenergic receptors stimulates cortical excitability and increases the concentration of DA (Smith, 2003; Minzenberg, 2012; Volkow and Swanson, 2013; Chabreck, 2015; Stewman et al., 2018; Berezanskaya et al., 2022). Long-term MPH treatment has been shown to decrease DAT density in the striatum, thereby increasing the transportation of vesicular DA *via* vesicular monoamine transporter-2 (VMAT-2), leading to more DA accumulating within the synapse (Smith, 2003; Minzenberg, 2012; Volkow and Swanson, 2013; Chabreck, 2015; Stewman et al., 2018; Berezanskaya et al., 2022). MPH can also increase the length and complexity of dendrites in cortical areas, increasing the responsivity of the prefrontal cortex neurons without changing prefrontal cortex discharge rates, and increasing the inhibitory component of the locus coeruleus (Smith, 2003; Minzenberg, 2012; Volkow and Swanson, 2013; Chabreck, 2015; Stewman et al., 2018; Berezanskaya et al., 2022). Similar to MPH, amphetamines inhibit DAT and NET reuptake of DA/NE into the presynaptic cell and increases extracellular concentrations of the neurotransmitters in multiple brain regions, and also decreases CBF (Smith, 2003; Minzenberg, 2012; Volkow and Swanson, 2013; Chabreck, 2015; Stewman et al., 2018; Berezanskaya et al., 2022). The increase in DA/NE concentrations within the synapse increases the incidence of receptor binding and since amphetamine inhibits monoamine oxidase, less DA/NE is removed from the synapse (Smith, 2003; Minzenberg, 2012; Volkow and Swanson, 2013; Chabreck, 2015; Stewman et al., 2018; Berezanskaya et al., 2022). Amphetamine is distinct from MPH due to the inhibition of VMAT-2 which causes the release DA/NE into the presynaptic terminal by presynaptic vesicles, and the internalization of a DAT resulting in the reversal of the transporter from in-slow to dual-flow of DA/NE reuptake. Therefore, amphetamine leads to a progressive increase in DA release that can be four times greater than the effects of MPH, in addition to increasing NE efflux by reversing the NET (Smith, 2003; Minzenberg, 2012; Volkow and Swanson, 2013; Chabreck, 2015; Stewman et al., 2018; Berezanskaya et al., 2022).

## Issues with attention-deficit/hyperactivity disorder pharmacotherapy

In recent years, misuse of stimulant medications for non-medical use has risen (White et al., 2014; Chabreck, 2015; Reardon and Factor, 2016; Poysophon and Rao, 2018; Stewman et al., 2018; Berezanskaya et al., 2022). Research has estimated a 4–14% yearly incidence of stimulant abuse in university students, and in a study with 115 undergraduate students, 31% indicated misuse to improve academics (White et al., 2014; Chabreck, 2015; Reardon and Factor, 2016; Poysophon and Rao, 2018; Stewman et al., 2018; Berezanskaya et al., 2022). Astonishingly, stimulant misuse is two times greater than the percentage of prescribed use and is higher than other drugs prevalence, such as cocaine, inhalants, and hallucinogens nationally, in young adults (White et al., 2014; Chabreck, 2015; Reardon and Factor, 2016; Poysophon and Rao, 2018; Stewman et al., 2018; Berezanskaya et al., 2022). There are two possible reasons why the misuse of stimulants has become more prevalent. First, some individuals use non-medical stimulants to self-medicate symptoms, most likely because they believe they have ADHD (White et al., 2014; Chabreck, 2015; Reardon and Factor, 2016; Poysophon and Rao, 2018; Stewman et al., 2018; Berezanskaya et al., 2022). Second, social stress, pressure, and anxiety to succeed in school and life have led to misuse due to medication increasing attention and longer academic study-periods (White et al., 2014; Chabreck, 2015; Reardon and Factor, 2016; Poysophon and Rao, 2018; Stewman et al., 2018; Berezanskaya et al., 2022).

The ethics of stimulant use in athletics has been a debated topic in sports performance since 1959 when a number of athletes admitted to stimulant use (Reardon and Factor, 2016). However, it’s increased in prominence within recent years due to increased ADHD diagnoses and recognition of the importance of mental disorders and mental health in the general population. Stimulants may confer advantages in a multitude of sports and enhance exercise performance (White et al., 2014; Chabreck, 2015; Reardon and Factor, 2016; Poysophon and Rao, 2018; Stewman et al., 2018; Berezanskaya et al., 2022). Many studies have shown increased performance in swimming, running, cycling, throwers, and aesthetic/weight class sports due to improved fine motor coordination, weight loss, enhanced fatigue perception, and attention (White et al., 2014; Chabreck, 2015; Reardon and Factor, 2016; Poysophon and Rao, 2018; Stewman et al., 2018; Berezanskaya et al., 2022). A recent meta-analysis Berezanskaya et al. (2022) found that the greatest effect sizes, regarding the effect of stimulants on performance outcomes, are within time-trial performance, power output, and knee extension strength. Studies assessing the ergogenic benefit of amphetamines on exercise performance have shown that, a 14–15 mg dose increased cycling time-trial performance and time to exhaustion, acceleration, and knee extensor strength (White et al., 2014; Chabreck, 2015; Reardon and Factor, 2016; Poysophon and Rao, 2018; Stewman et al., 2018; Berezanskaya et al., 2022). For those taking 20–21 mg of MPH, cycling time-trial performance was completed 16% faster, power output increased, and a greater mean force in hand grip exercise was observed compared to placebo (White et al., 2014; Chabreck, 2015; Reardon and Factor, 2016; Poysophon and Rao, 2018; Stewman et al., 2018; Berezanskaya et al., 2022).

Athletes with ADHD that are taking stimulants must submit documentation to the World Anti-Doping Agency (WADA) and the National Collegiate Athletic Association (NCAA) to receive a Therapeutic Use Exemption (TUE) (White et al., 2014; Chabreck, 2015; Reardon and Factor, 2016; Poysophon and Rao, 2018; Stewman et al., 2018; Berezanskaya et al., 2022). Documentation for a TUE must contain a description of ADHD assessment, evaluation tools/procedures used, diagnosis statement, history of previous and current ADHD diagnoses and treatment, (for NCAA) a statement of a non-stimulant alternative, and a past and current plan for follow-up and monitoring visits (White et al., 2014; Chabreck, 2015; Reardon and Factor, 2016; Poysophon and Rao, 2018; Stewman et al., 2018; Berezanskaya et al., 2022). Physicians have several options that they can use to prescribe stimulant treatment to athletes. One option is the timing of medication, specifically for performance in academics and practice, and not competition (White et al., 2014; Chabreck, 2015; Reardon and Factor, 2016; Poysophon and Rao, 2018; Stewman et al., 2018; Berezanskaya et al., 2022). Another option is medication that lasts for an extended period for performance in academics, practice, and competition (White et al., 2014; Chabreck, 2015; Reardon and Factor, 2016; Poysophon and Rao, 2018; Stewman et al., 2018; Berezanskaya et al., 2022). However, athletes may prefer one approach over another depending on type of sport they participate in and/or sport position, and what they perceive to be best for their athletic performance. For example, some athletes may perform better without medication because of spontaneity or unpredictability (i.e., football). In contrast, others may perform better with medication because of improved focus and decision-making (i.e., tennis) (White et al., 2014; Chabreck, 2015; Reardon and Factor, 2016; Poysophon and Rao, 2018; Stewman et al., 2018; Berezanskaya et al., 2022).

## Non-pharmacological alternatives for attention-deficit/hyperactivity disorder

Not all ADHD patients or athletes choose stimulant medication for treatment due to personal, financial, or other reasons. One option is non-stimulant medication such as atomoxetine and behavioral and specialized therapy. Another option is the implementation of exercise, and the potential effects of caffeine supplementation.

## Neurobehavioral, cognitive, and neurophysiological effects of exercise in ADHD

Exercise is a powerful non-pharmacological ADHD treatment since it can increase cerebral blood flow, brain activation, DA/NE concentrations, and elevate arousal to yield beneficial cognitive effects on extraneous stimuli inhibition, ability to move deliberately between different tasks, and sustained attention capability (Volkow and Swanson, 2013; Chabreck, 2015; Choi et al., 2015; Gapin et al., 2015; Connell et al., 2016; Reardon and Factor, 2016; Rassovsky and Alfassi, 2018; Huertas et al., 2019; Mehren et al., 2019). Gapin et al. (2015) sought to identify the impact of 30-minute treadmill exercise performed at 50–65% of heart rate reserve, on cognitive functions in college students with and without ADHD. Prior to exercise, ADHD students displayed deficits for all three administered cognitive tests, but only the color-word condition of the Stroop Test was different from the non-ADHD students (Gapin et al., 2015). After exercise, the non-ADHD students improved in all three cognitive tasks, while the ADHD students improved only in the Stroop tests’ cognitive assessment. These data demonstrate that exercise increases attention span, stimulus selection speed, and improves inhibition of unrelated stimuli (Gapin et al., 2015). Rassovsky and Alfassi (2018) looked at continuous auditor attentional tests during 14-minute treadmill exercise at 5°km/h to measure changes in sustained attention in ADHD participant’s verses controls. They found a significant group by exercise interaction, whereby the ADHD participants improved reaction times and reduced errors while exercising compared to their resting scores and the non-ADHD participants. The data suggest that treadmill exercise improved stimuli inhibition which potentially changed how the participant evaluated task and attentional stimuli (Rassovsky and Alfassi, 2018). Choi et al. (2015) examined children/adolescent participants with ADHD taking MPH and the effect of aerobic exercise on cognitive tasks. Participants were randomized into sport-ADHD and education-ADHD groups, where the sports group completed 60-minutes of varying activities (i.e., shuttle run, jump rope, and basketball) 3-times a week, and the education group completed a similar amount of time dedicated to schoolwork. Exercise increased MPH effectiveness as evidence by decreased symptoms, errors, and increased brain activity in response to working memory tasks in the right frontal cortex and parietal regions. In addition, right temporal lobe activity during worming memory tasks decreased after 6 weeks of exercise, representing increased attentional processing speed (Choi et al., 2015). Lastly, Mehren et al. (2019) showed that after moderate intensity exercise (50–70% HR_max_), ADHD participants with the worst cognitive performance showed improvements in executive function, which was associated with increased brain activity in task-related regions.

Decreases in CBF in ADHD participants compared to controls in the somatomotor, ventral attentional, and limbic networks have been consistently reported in ADHD (Ogoh and Ainslie, 2009; Pires et al., 2018; Franco-Alvarenga et al., 2019; Tan et al., 2020). Tan et al. (2020) found significant negative correlations between CBF and ADHD symptoms, where the greatest decrease in CBF increased the presence of ADHD symptoms. A few studies have demonstrated that ADHD individuals participating in low to moderate exercise improve cognitive performance due to increased brain activation in the frontoparietal regions and increased CBF (Gapin et al., 2015; Rassovsky and Alfassi, 2018; Tan et al., 2020). In healthy participants, studies have shown increases in CBF during exercise (up to 60% of maximal oxygen consumption), however, following high-intensity exercise, CBF decreases in prefrontal, and frontal regions due to changes in CO_2_ partial-pressures, decreased glucose metabolism, and vasoconstriction (Ogoh and Ainslie, 2009; Pires et al., 2018; Franco-Alvarenga et al., 2019). One may postulate that decrements in CBF and brain activation at high-intensity exercise may promote increased central fatigue, and decrease exercise performance, in ADHD athletes compared to non-ADHD athletes. Overall, numerous studies (Xie et al., 2021) have shown that exercise is a beneficial supplement to medication or could be considered an alternative treatment plan to manage ADHD symptoms and improve cognitive performance and brain activation during and after exercise (Volkow and Swanson, 2013; Chabreck, 2015; Choi et al., 2015; Gapin et al., 2015; Connell et al., 2016; Reardon and Factor, 2016; Rassovsky and Alfassi, 2018; Huertas et al., 2019; Mehren et al., 2019).

## Neurobehavioral, cognitive, and neurophysiological effects of caffeine in attention-deficit/hyperactivity disorder

**FIGURE 2:**
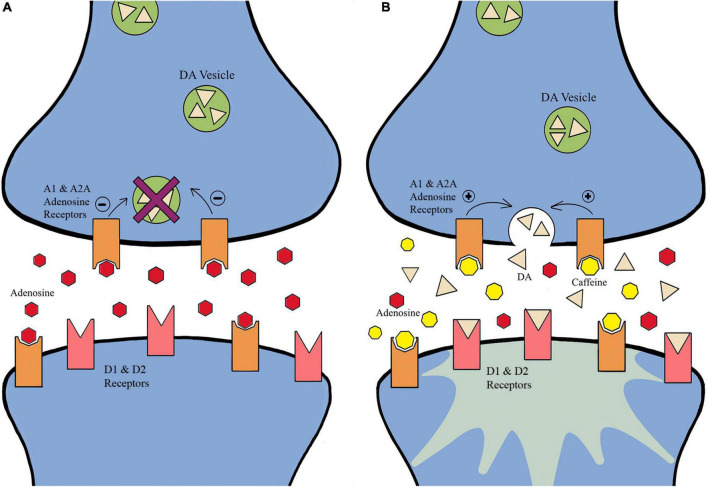
The effects of caffeine on adenosine receptors. **(A)** Adenosine binds to adenosine receptors and inhibits dopamine activity. **(B)** Caffeine acts as an antagonist where it blocks adenosine binding to adenosine receptors, enhancing dopamine activity. The mechanism increases neuron excitability, promotes neurotransmitter release, increase brain activity, and may improve spinal and supraspinal excitability (Fredholm et al., 1999; Smith, 2003; Hogervorst et al., 2008; Connell et al., 2016; Pires et al., 2018; Saville et al., 2018; Shabir et al., 2018; Franco-Alvarenga et al., 2019; Huertas et al., 2019; Cipollone et al., 2020; Machado et al., 2020; Sanchis et al., 2020; Wang et al., 2020; Lorenzo Calvo et al., 2021; Vázquez et al., 2022; Ágoston et al., 2022).

Caffeine (CAF; 1,3,7-trymethylxanthine) is the most available and highly consumed substance within the global population (Smith, 2003; Hogervorst et al., 2008; Connell et al., 2016; Pires et al., 2018; Saville et al., 2018; Shabir et al., 2018; Franco-Alvarenga et al., 2019; Huertas et al., 2019; Cipollone et al., 2020; Machado et al., 2020; Sanchis et al., 2020; Wang et al., 2020; Lorenzo Calvo et al., 2021; Vázquez et al., 2022; Ágoston et al., 2022). Once a banned substance within sport, in 2004, CAF became a monitored substance and has reached a prevalence rate of 76% within international competition (Smith, 2003; Hogervorst et al., 2008; Connell et al., 2016; Pires et al., 2018; Saville et al., 2018; Shabir et al., 2018; Franco-Alvarenga et al., 2019; Huertas et al., 2019; Cipollone et al., 2020; Machado et al., 2020; Sanchis et al., 2020; Wang et al., 2020; Lorenzo Calvo et al., 2021; Vázquez et al., 2022; Ágoston et al., 2022). Recommended dosages for CAF fall between 3 and 6 mg/kg body weight and a possible upward dose of 9 mg/kg (Smith, 2003; Hogervorst et al., 2008; Connell et al., 2016; Pires et al., 2018; Saville et al., 2018; Shabir et al., 2018; Franco-Alvarenga et al., 2019; Huertas et al., 2019; Cipollone et al., 2020; Machado et al., 2020; Sanchis et al., 2020; Wang et al., 2020; Lorenzo Calvo et al., 2021; Vázquez et al., 2022; Ágoston et al., 2022). CAF affects many aspects of human performance such as alertness, concentration, energy level, self-reported fatigue, substrate use for energy provision, reaction time, and error rate in simple and complex cognitive tasks (Smith, 2003; Hogervorst et al., 2008; Connell et al., 2016; Pires et al., 2018; Saville et al., 2018; Shabir et al., 2018; Franco-Alvarenga et al., 2019; Huertas et al., 2019; Cipollone et al., 2020; Machado et al., 2020; Sanchis et al., 2020; Wang et al., 2020; Lorenzo Calvo et al., 2021; Vázquez et al., 2022; Ágoston et al., 2022). The ergogenic effects of CAF on aerobic performance are usually observed 60-minutes after ingestion when CAF reaches peak levels in blood, followed by an increase in lipolysis and efficient usage of glucose *via* phosphofructokinase (Smith, 2003; Hogervorst et al., 2008; Connell et al., 2016; Pires et al., 2018; Saville et al., 2018; Shabir et al., 2018; Franco-Alvarenga et al., 2019; Huertas et al., 2019; Cipollone et al., 2020; Machado et al., 2020; Sanchis et al., 2020; Wang et al., 2020; Lorenzo Calvo et al., 2021; Vázquez et al., 2022; Ágoston et al., 2022). Additionally, CAF peripherally is beneficial within high-velocity movement sports due to blocking adenosine receptors in the neuromuscular region and increasing the effect of the sodium-potassium ATPase pump, resulting in an increase in the depolarization of the skeletal muscle T-tubules, and enhancing the release of calcium from the sarcoplasmic reticulum. Furthermore, the increased calcium concentrations in the muscle cytoplasm, increases calcium binding opportunities for troponin to expose myosin binding sites and thus enhance skeletal muscle contraction force (Smith, 2003; Hogervorst et al., 2008; Connell et al., 2016; Pires et al., 2018; Saville et al., 2018; Shabir et al., 2018; Franco-Alvarenga et al., 2019; Huertas et al., 2019; Aguiar et al., 2020; Cipollone et al., 2020; Machado et al., 2020; Sanchis et al., 2020; Wang et al., 2020; Lorenzo Calvo et al., 2021; Vázquez et al., 2022; Ágoston et al., 2022). Centrally, the most accepted mechanism of how CAF exerts its effect on cognition is its influence on the central neuronal adenosine receptors, specifically the A_1_ and A_2A_ adenosine receptors (Fredholm et al., 1999; Dunwiddie and Masino, 2001; Smith, 2003; Borycz et al., 2007; Hogervorst et al., 2008; Pandolfo et al., 2013; Connell et al., 2016; Cunha, 2016; Leffa et al., 2018, 2019; Pires et al., 2018; Saville et al., 2018; Shabir et al., 2018; Franco-Alvarenga et al., 2019; Huertas et al., 2019; Lopes et al., 2019; Aguiar et al., 2020, 2021; Cipollone et al., 2020; Machado et al., 2020; Sanchis et al., 2020; Wang et al., 2020; Lorenzo Calvo et al., 2021; Vázquez et al., 2022; Ágoston et al., 2022). Extracellular adenosine in the brain is formed by AMP-selective enzymes, such as CD73, and is dependent on the rate of synthesis and breakdown of ATP (Aguiar et al., 2021). CAF is an adenosine antagonist whereby it blocks adenosine binding to A_1_ and A_2A_ receptors by reducing adenosine’s ability to inhibit DA activity. This mechanism increases neuron excitability, promotes neurotransmitter release, increases brain activity, influences information processing, locomotion control, and arousal, and may improve spinal and supraspinal excitability (Figure 2; Fredholm et al., 1999; Smith, 2003; Ferré, 2008; Hogervorst et al., 2008; Pandolfo et al., 2013; Connell et al., 2016; Leffa et al., 2018, 2019; Pires et al., 2018; Saville et al., 2018; Shabir et al., 2018; Franco-Alvarenga et al., 2019; Huertas et al., 2019; Lopes et al., 2019; Aguiar et al., 2020; Cipollone et al., 2020; França et al., 2020; Machado et al., 2020; Sanchis et al., 2020; Wang et al., 2020; Lorenzo Calvo et al., 2021; Vázquez et al., 2022; Ágoston et al., 2022). For example, in the hippocampus of mice, a moderate dose of caffeine was shown to impact the transmission and plasticity of excitatory synapses, which was selectively dependent on the presence of extracellular adenosine at A_1_ and A_2A_ receptors (Lopes et al., 2019).

Caffeine has the ability to control the density and activity of DAT, namely in ADHD animal models. Pandolfo et al. (2013) tested if chronic caffeine ingestion could improve certain characteristics of ADHD. In a hypertensive rat model of ADHD, they found that chronic caffeine ingestion (2°mg/kg) in ADHD rats negates neuronal circuit dysfunction, normalizes DAT density and function, and showed that the reversal of behavioral deficits in the frontal cortex related to sustained attention, all mediated by the A_2A_ receptors. Additionally, DA uptake was significantly reduced after chronic caffeine ingestion in the ADHD rat model, which reflects the normalization of DAT, leading to an increase in DA within the synapse for a longer period of time.

Pandolfo et al. (2013) also demonstrated an increase in A_2A_ receptors in the frontocortical nerve terminals in the ADHD rat, indicating that A_2A_ receptors serve a prominent role in ADHD circuitry and possibly behavioral deficits. In support of this notion, Leffa et al. (2018) examined the effects of A_2A_ receptor downregulation in the prefrontal cortex (PFC) on different behavioral aspects such as delayed-based cost-benefit decision-making to measure impulsivity. The data indicate an increase in impulsive behavior due to the downregulation of A_2A_ receptors in the PFC, which influenced the control of delayed-based cost-benefit decision-making (Leffa et al., 2018, 2019). With this in mind, similar animal studies have shown that caffeine can possibly reduce impulsivity in rats with a medium to high basal impulsivity level (Waldeck and Miller, 1997; Fredholm et al., 2005; Jones and Lejuez, 2005; Barbelivien et al., 2008; Cunha and Agostinho, 2010; Chen, 2014; Ruiz-Oliveira et al., 2019), due to the antagonism of adenosine receptors resulting in the normalization of impulsive function, such that already elevated impulsivity will be dampened while low basal impulsivity is improved. Furthermore, A_2A_–D_2_ heteromeric interactions are increasingly recognized as being paramount in the control of dopaminergic signaling and behavior (Ferré, 2008; Pandolfo et al., 2013). It has been shown that (Ferré, 2008; Pandolfo et al., 2013) stimulation of the A_2A_ reduces D_2_ dopamine binding, thus controlling neurotransmitter release, and neuronal excitability and firing, by depressing D_2_ receptor stimulation. However, it has been shown that antagonistic agents (CAF) interact between A_2A_ and D_2_, and stimulate the D_2_ receptor which depresses A_2A_ receptor stimulation, and plays a primary role in motor activation, resulting in downstream positive effects for L-DOPA (Ferré, 2008; Pandolfo et al., 2013).

Since a large portion of the general population consumes CAF and has been habituated to CAF-use to some degree, habituated consumers may have to take greater amounts of CAF to induce comparable dopaminergic effect as observed in low to non-habituated users (Smith, 2003; Hogervorst et al., 2008; Connell et al., 2016; Pires et al., 2018; Saville et al., 2018; Shabir et al., 2018; Franco-Alvarenga et al., 2019; Huertas et al., 2019; Cipollone et al., 2020; Machado et al., 2020; Sanchis et al., 2020; Wang et al., 2020; Lorenzo Calvo et al., 2021; Vázquez et al., 2022; Ágoston et al., 2022). It is postulated in humans, and shown in multiple animal studies that acute and chronic CAF ingestion selectively antagonizes adenosine receptors (Ferré, 2008; Pandolfo et al., 2013; Cunha, 2016; Leffa et al., 2019; França et al., 2020). Acute ingestion of CAF mostly acts through the antagonism of the A_1_ receptors, leading to increases in excitability but results in less neuroprotection (Cunha, 2016). Whereas chronic ingestion mostly acts through the antagonism of A_2A_ receptors, affording neuroprotection by preventing memory and motor deficits (Cunha, 2016).

Numerous animal studies have examined the effect of CAF consumption or CAF ingestion combined with and exercises on ADHD-like symptoms. Caballero et al. (2011) found that after 14 days of caffeine ingestion, neonatal 6-hydroxyl-dopamine lesioned rats (an animal model for ADHD) significantly improved their attention deficit during the prepubertal period of ADHD. Higgins et al. (2007) has shown that 3–10°mg/kg of caffeine ingestion in rats increased reaction times in a selective attention task, where the 3°mg.kg caffeine dose produced faster responses while the 10°mg/kg dose increased the number of premature responses. França et al. (2020) found that in a rat animal model of ADHD combining caffeine (0.3°mg/mL in drinking water) and exercise for 6 weeks did not change spontaneous locomotion in an open field test. However, the association of exercise with caffeine improved working memory and that caffeine with exercise improved olfactory deficits. Importantly, it was found that caffeine and exercise, on its own or combined, increased striatal DA levels (França et al., 2020). While Szczepanik et al. (2016); Pires et al. (2010), and Prediger et al. (2005) demonstrated that caffeine ingestion does not change locomotor activity in animal models of ADHD, Prediger et al. (2005) has shown that pre-training caffeine ingestion (1–10°mg/kg) improved spatial learning, and Leffa et al. (2019) showed an increase in the number of large-reward choices in ADHD rats after 2 or 5°mg/kg pretreatment of caffeine. However, a 2°mg/kg dose of CAF for 21 days increased impulsivity and decreased large-reward choices. Nunes et al. (2018) found improved recognition memory in male and female rates following caffeine ingestion (0.3°g/L). Collectively, it has been shown that CAF increases cognitive performance such as attention, memory, and learning at the molecular and neuronal level, which suggest that the cognitive improvements found in animal studies of ADHD may be conveyed to humans with ADHD (Vázquez JC).

In the non-ADHD human population, many studies have been conducted regarding CAF’s effects on cognitive performance. In many cases, CAF doses of 2.5–9°mg/kg or 100–500°mg have been shown to affect the processing of stimuli, decision making, attention, reaction times, cognitive visual processing, and executive function during exercise, and reduced error rates either during or after exercise after an hour following ingestion (Smith, 2003; Oei and Hartley, 2005; Hogervorst et al., 2008; Harrell and Juliano, 2009; Connell et al., 2016; Pires et al., 2018; Saville et al., 2018; Shabir et al., 2018; Franco-Alvarenga et al., 2019; Huertas et al., 2019; Machado et al., 2020; Sanchis et al., 2020; Wang et al., 2020; Lorenzo Calvo et al., 2021). Huertas et al. (2019) and Hogervorst et al. (2008), showed that after consumption of 4°mg/kg and 100°mg of CAF respectively, attention and response speed/reaction time improved as well as visual processing during cognitive tasks that were more complex (i.e., rapid visual processing test and visual search test) than simple (i.e., color-word test and word learning test). In addition, Huertas et al. (2019) showed differences in consumer habituation, where high consumers displayed reduced alertness at rest compared to exercise, while the low consumer’s reaction speed to presented stimuli was reduced only during exercise. These data suggest that high consumers may need greater doses of CAF to induce similar changes seen in the low consumers to improve cognitive performance (Hogervorst et al., 2008; Huertas et al., 2019). A study conducted by Connell et al. (2016) also demonstrated improvements in reaction times with two, 2.5°mg/kg CAF doses, given throughout 180 min of cycling exercise at a predetermined power output, although spatial attention was unaffected. Comparing doses of 3, 6, and 9°mg/kg on cognitive performance, Wang et al. (2020) found that low doses of CAF improved the Stroop cognitive tests and reaction times, and low to moderate doses decreased cognitive interference, because fewer stimuli interfered with processing and decision making. Interestingly, studies have shown decreases in prefrontal cortex activation, with no changes in motor cortex activation, and changes in prefrontal cortex deoxygenation being present when assessing cognitive performance during and after exercise at moderate to submaximal intensities (Pires et al., 2018; Franco-Alvarenga et al., 2019). However, the decreases in activation and greater deoxygenation during exercise in the CAF participants (5–6°mg/kg) did not affect muscular activity or motor output (Pires et al., 2018; Franco-Alvarenga et al., 2019). This observation may be due to CAF’s effects on the central nervous system such that increased central neuronal efficiency and less excitatory input from the motor cortex may maintain muscular motor output (Pires et al., 2018; Franco-Alvarenga et al., 2019).

The effects of CAF supplementation on children with ADHD have been widely studied. Several studies have shown that (Garfinkel et al., 1975; Schecter and Timmons, 1985; Smith, 2003) the greatest reduction in cognitive task errors occurs in high-amphetamine treatment group, but the CAF and low-amphetamine treatment group results in the greatest overall decrease in errors, suggesting an enhanced ability to filter and process information (Garfinkel et al., 1975; Schecter and Timmons, 1985; Smith, 2003). Additionally, it has been demonstrated that a dose of CAF greater than ∼160°mg worsened ADHD symptomology with MPH medication (Garfinkel et al., 1975; Schecter and Timmons, 1985; Smith, 2003). Thus, a low dose of CAF with MPH would be the most effective supplementation approach. Studies examining caffeine supplementation in ADHD adults are scarce and equivocal (Garfinkel et al., 1975; Schecter and Timmons, 1985; Smith, 2003). In adult ADHD, Cipollone et al. (2020) found a few small positive correlations between CAF and ADHD indicating CAF as a possible supplement to help ADHD adults with everyday cognitive and functional tasks. Conversely, Ágoston et al. (2022) found that CAF consumption did not correlate with several ADHD symptoms. Therefore, while CAF has the potential to be of benefit to adults with ADHD, more studies need to be conducted.

## Conclusion and future directions

Attention-deficit/hyperactivity disorder diagnosis rates have continued to increase within the recent decade, with many diagnoses continuing well into adulthood. While the efficacy of exercise in managing ADHD symptoms is well known, the use of CAF alone and with exercise is potentially impactful, and therefore should be considered in the treatment plan for an individual with ADHD. However, studies examining the effects of CAF in adult ADHD athletes are lacking. Future research should examine the effects of caffeine on an ADHD athlete’s exercise and cognitive performance with and without medication, and to better understand the effects of ADHD on central fatigue.

## Author contributions

Both authors contributed to the overall collection of information and writing and final revision of the manuscript and approved the submitted version.
